# Nucleation increases the visual appeal of lager but does not alter overall likeability or drinking rate

**DOI:** 10.1186/s12954-022-00618-4

**Published:** 2022-04-20

**Authors:** David M. Troy, Olivia M. Maynard, Matthew Hickman, Marcus R. Munafò, Angela S. Attwood

**Affiliations:** 1 School of Population Health Sciences, University of Bristol, Canynge Hall, 39 Whatley Road, BS8 2PS Bristol, UK; 2 MRC Integrative Epidemiology Unit (IEU) at the University of Bristol, Bristol, UK; 3 School of Psychological Science, University of Bristol, Bristol, UK; 4 NIHR Biomedical Research Centre at the University Hospitals NHS Foundation Trust, Bristol, UK

**Keywords:** Nucleation, Likeability, Drinking rate, Glassware

## Abstract

**Background:**

Glassware can be an effective vehicle to recruit customers, revive brands, build profits and increase alcohol consumption by capitalising on the immediacy of glassware to the point of consumption. The design of glassware can also contribute to harm reduction by slowing and reducing consumption. Nucleated bases have been added to lager glasses in recent years which allow carbon dioxide (CO_2_) to be more rapidly released and ascend through the solution. The aim of these studies was to investigate the effect of nucleated glasses on the likeability and drinking rate of lager in alcohol drinkers.

**Methods:**

In Study 1, participants (*n* = 116) were asked to taste two glasses of lager (280 millilitres (ml) each) in separate 5-min taste tests and fill out a likeability questionnaire after each glass in a within-subjects design with one factor of glass (nucleated, non-nucleated). The primary outcome was the likeability of lager and the secondary outcome was volume consumption during taste tests. In Study 2, participants (*n* = 160) were asked to consume a pint of lager (568 ml) and fill out a likeability questionnaire in a between-subjects design with one factor of glass (nucleated, non-nucleated). The primary outcome was time taken to consume a pint of lager and secondary outcomes were the likeability of lager, mood and alcohol craving.

**Results:**

There was no clear evidence that likeability of lager differed between nucleated and non-nucleated glasses in either study. In Study 1, a paired-samples *t* test found strong evidence that lager in nucleated glasses was more visually appealing (single item from likeability measure) than lager in non-nucleated glasses (mean difference (MD) = 10.2, 95% confidence interval (CI) 6.1, 14.2, *p* < 0.001). In Study 2, a linear regression found no clear evidence that lager was consumed at different rates from nucleated and non-nucleated glasses (nucleated: 16.9 min, non-nucleated: 16.3 min, MD: 0.6 min, 95% CI − 1.5, 2.7, *p* = 0.57).

**Conclusions:**

Nucleated lager glasses do not appear to alter the likeability or consumption (volume consumed in Study 1 or drinking rate in Study 2) of lager, although they do seem to increase the visual appeal and refreshment of lager. This may increase the number of drinking episodes by making the drinking experience more enjoyable which may lead to increased alcohol related harm.

**Supplementary Information:**

The online version contains supplementary material available at 10.1186/s12954-022-00618-4.

## Introduction

The World Health Organization (WHO) defines alcohol harm reduction as “policies or programmes that focus directly on reducing the harms from the use of alcohol” [1]. The WHO proposes ten target areas for national policy development to reduce alcohol harms, nine of which target reduced consumption via supply or demand side mechanisms. The remaining area focuses on “reducing the harm from alcohol intoxication and drinking without necessarily affecting the underlying consumption” [2]. Glassware is one target in a drinking environment that can be altered to change alcohol behaviours and potentially reduce alcohol related harms [3]. The alcohol industry utilises glassware as an effective vehicle to recruit customers, revive brands, build profits and increase consumption by capitalising on the immediacy of glassware to the point of consumption [4]. The public health community has been increasingly researching choice architecture interventions in an attempt to nudge consumers into engaging in healthier behaviours [5]. A recent development in glassware is the addition of nucleated bases in lager glasses. Research is needed to establish what effect this design feature has on the likeability and drinking rate of lager, as this could have implications for harm reduction measures and/or policies in terms of reducing demand for lager products and/or reducing intoxication due to slower consumption.

Nucleation is a process in supersaturated solutions whereby gases such as CO_2_ are released. Bubbles of CO_2_ molecules grow on nucleation sites which usually come in the form of hollow, cylindrical cellulose fibres [6, 7] and are released from these sites when they reach a critical size and ascend through the solution. Bubbles rapidly grow in size as they ascend and increase in speed as they travel upward [8]. Modern lager glasses concentrate the nucleation process by having either a laser-etched or printed nucleated stamp on the inner base, which allows CO_2_ to be more rapidly released.

Few studies have examined correlates of nucleation such as the head of a beer (which can be maintained for longer by nucleation) and CO_2_ content (which can be increased by nucleation). Beer with a sizable head has been judged to taste better than a beer with less head [9]. Italian consumers concluded that beer with a medium (compared to larger or smaller) level of foam was the best dispensed, most visually appealing, most attractive to consume and most likely to be purchased [10]. Beers of higher CO_2_ content have been perceived as more bitter [11, 12], and CO_2_ has been deemed to have an important role in conveying beer flavour, aroma delivery and mouth feel [13, 14].

The effect of nucleation on the drinking experience of champagne and other sparkling wines has been studied more extensively and can inform our understanding of the experience of consuming a nucleated lager. Nucleation in sparkling wines produce rising bubbles that impact the visual perception of wine before the act of tasting and inhaling has begun [6]. The aromatic perception of sparkling wine is due to bursting bubbles releasing gaseous CO_2_ and volatile organic compounds above the wine surface [15, 16]. Dissolved CO_2_ and collapsing bubbles in the oral cavity interact with trigeminal receptors which are responsible for face sensations [17, 18] and gustatory receptors which are responsible for taste sensations [19, 20]. These reactions may influence a lager drinker in similar ways. The influence of glassware on consumer perceptions of the contents appears to be primarily psychological rather than chemical or physical in origin [21] as perceptible differences largely disappear when participants taste the same wine sample in different shaped glasses when their focus is on the contents and not the glassware [22, 23]. It is unclear what influence nucleating a lager glass will have on the psychological processes at work during lager consumption.

Glassware is a modifiable vehicle for the alcohol industry to influence how their products are perceived and consumed. Various characteristics of glassware such as the weight, size, shape and colour can influence the taste and/or flavour of wine and beer (e.g. [24, 25]). Glass shape can also affect consumption of alcoholic beverages as straight sided glasses have resulted in slower lager drinking when compared to curved glasses [26] possibly due to more accurate volume perceptions when drinking from straight glasses. In support, a study has shown that volume judgement when pouring is more accurate in straight glasses compared to curved glasses [27]. Perhaps even more relevant for this study, a more nuanced change to glassware when volume markers (at ¼, ½ & ¾) were applied to curved glasses slowed lager consumption when compared to unmarked curved glasses [28]. These alterations of glassware could potentially lead to a reduction in social harms associated with excessive alcohol consumption due to decreased intoxication from slower consumption and a reduction in health harms due to a reduction in overall consumption if fewer alcoholic beverages are consumed per drinking episode. Similarly, nucleating of lager glasses may also affect the consumption of lager which may increase alcohol related harm.

In summary, “head” and CO_2_ content, which are altered by nucleation, appear to affect the sensory experience of consuming lager. In Study 1, we investigated the effect of nucleated glasses on self-reported likeability of lager and amount consumed in a 5 min period. We hypothesised that lager in nucleated glasses would be rated as more likeable than lager in non-nucleated glasses. In Study 2, we investigated the effect of nucleated glasses on the likeability and drinking rate of lager. We hypothesised that there would be a difference in drinking rate between the glasses, but this was a non-directional hypothesis. If the likeability of lager in nucleated glasses is greater than in non-nucleated glasses, this may speed up consumption due to a more pleasant and rewarding drinking experience. In contrast the increased effervescence may lead consumers to savour the more likeable drinking experience and be less concerned with finishing the drink before it goes “flat”.

## Methods

### Study 1

#### Design and overview

This was a laboratory-based experimental study. We used a within-subjects, double-blind design with one factor of glass type (nucleated, non-nucleated). The presentation order of the glasses was counterbalanced, and each condition was populated with an equal number of participants stratified by sex. A study protocol detailing data collection procedures, data analysis, data management, etc. was registered on the Open Science Framework (http://osf.io/yzvk5) prior to data collection.

#### Participants

Alcohol drinkers who reported consuming between 10 and 50 units/week if male or between 5 and 35 units/week if female (one unit is 10 ml or 8 g of pure alcohol) were recruited from the staff and students of the University of Bristol and from the general population by means of poster and flyer advertisements, existing database of participants who regularly participate in research and word of mouth. Participants were required to be in good psychological and physical health, aged between 18 and 40 years, and not currently taking any psychiatric medication. Exclusion criteria included current use of illicit substances (excluding cannabis), a strong family history of alcoholism (defined as at least one first-degree relative or two or more second degree relatives), weighing less than 50 kg (kg) if female or 60 kg if male and not drinking/liking lager. Participants were asked to abstain from alcohol consumption for 24 h prior to the test session and were only enrolled onto the study if they provided a zero breath alcohol concentration reading at the start of the session. Participants were reimbursed £5 or awarded course credit at the end of the study.

#### Materials

**Fig. 1:**
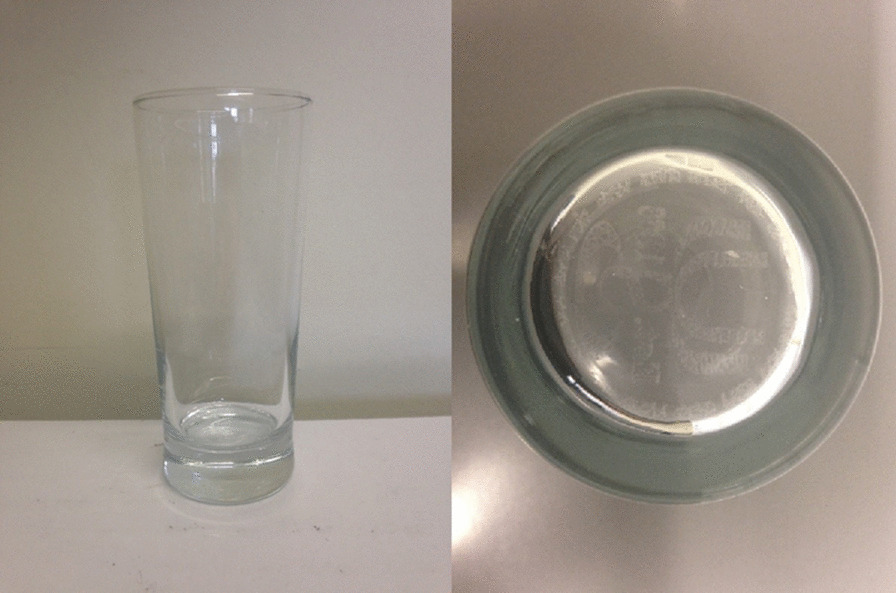
Senator beer glass (left) with its nucleated base (right)

The alcoholic beverage used was lager (Budweiser™ 4.8% alcohol by volume [ABV]). Budweiser was chosen as it is a standard strength, commonly consumed lager in the UK. Glassware used were Senator beer glasses (volume: 280 ml; Fig. 1) designed by Paşabahçe*.* One was a “Super Activator Max” nucleated glass, and the other was non-nucleated. The glasses were identical in all other respects.

Questionnaire measures comprised the Alcohol Use Disorder Identification Test (AUDIT) [29] to assess drinking patterns and a Lager Drinking Experience Questionnaire (LDEQ) to assess likeability of lager amended from a taste test questionnaire used with permission from colleagues at the University of Liverpool [30]. The LDEQ contains ten questions (“How smooth is this drink?”, “How light is this drink?”, “How sweet is this drink?”, “How intoxicating is this drink?”, “How bubbly/gassy is this drink?”, “How visually appealing is this drink?”, “How enjoyable is his drink?”, “How refreshing is this drink?”, “How tasty is this drink?”, “How likely would you be to buy this drink?”) which were rated on a 100 mm (mm) VAS from “Not at All” to “Extremely”.

#### Procedure

Participants attended one study session lasting approximately 30 min. Participants were sent the information sheet in advance of the study session and were given the opportunity to read it again upon arrival and ask questions. They were informed that the study was interested in determining how different factors such as the time taken to ferment a lager, the temperature it is set to and how strain of lager yeast used contribute to the look and taste of lagers. After informed consent had been obtained, a screening procedure was conducted to assess eligibility for the study, based on inclusion/exclusion criteria. Recent alcohol consumption was assessed by breath test (AlcoDigital 3000, UK Breathalysers) and weight was recorded.

For the main session, participants were asked to turn their phone off and place it out of reach. They were presented with 100 ml of water as a thirst quencher and told to consume as much as they liked. Baseline testing begun with participants completing the AUDIT. Whilst the AUDIT was being completed, 280 ml of lager was poured into a glass (either nucleated or non-nucleated glass as per randomisation) by a second experimenter (to maintain blinding) in a nearby kitchen and delivered to the test room. Drinks were chilled prior to serving and were poured immediately prior to consumption to ensure that carbonation was consistent across participants. The second experimenter presented the drink to the participant and the primary experimenter instructed the participant to consume as much of the drink as they wanted over a duration of 5 min, complete the LDEQ whilst doing so and place the glass in an adjacent box (to maintain blinding) when finished. The drinking phase started after the primary experimenter said: “You may begin”, and was recorded by stopwatch. The primary experimenter left the room for 5 min and then returned with another 100 ml of water for the participant to cleanse their palette. Participants were then given a magazine, and a 5-min break commenced. The primary experimenter returned to the room after the 5-min break was over. The second experimenter prepared another 280 ml of lager (either nucleated or non-nucleated glass as per randomisation) and delivered it to the test room. The procedure followed for the first drink was repeated and the same instructions were given. After the drink was consumed, participants were asked did they prefer Drink 1 or Drink 2.

Before leaving the testing room, participants were asked to read and sign a safety form that advised them that they had received alcohol and that they should not drive, cycle, operate machinery or engage in any other task or behaviour considered potentially hazardous after alcohol consumption. Participants were debriefed and reimbursed. Participants were offered the opportunity to stay behind to allow any effects of alcohol to wear off and a taxi home. When the participant left, the primary experimenter measured the remaining volume from the first and second drink (the participant was naïve to this).

#### Statistical analysis

A previous study investigating the effect of beverage packaging on the palatability of alcoholic beverages (no evidence from a study directly investigating nucleation was available) indicated an effect size from standardised difference scores (d*z*) of 0.27 (given a correlation of *r* = 0.74 between responses in the two conditions) for the difference in the palatability ratings of beer between a blind and non-blind condition [31]. To detect the same effect size, we required a sample size of 110 in order to achieve 80% power at an alpha level of 5%. This was increased to 112 participants to allow for equal numbers of males and females in each glass condition.

Questionnaire responses were captured via online survey platforms (Bristol Online Survey & Qualtrics) and imported into Statistical Package for the Social Sciences (SPSS). Volume consumption data was extracted from case report forms. Data from five questionnaire items in the LDEQ (“How visually appealing was the drink?”; “How enjoyable was the drink?”; “How refreshing was the drink?”; “How tasty was the drink?”; “How likely would you be to buy the drink?”) were averaged to calculate a likeability score. Other questions (“How smooth was the drink?”, “How light was the drink?”, “How sweet was the drink?”, “How intoxicating was the drink?”) acted as filler questions and were not analysed.

The primary outcome was the likeability of lager in nucleated and non-nucleated glasses analysed using a paired-samples *t*-test. Secondary outcomes were the volume consumed from each glass condition and the responses to the individual questionnaire items that constituted the likeability factor for each glass condition. These were analysed individually using paired-samples *t* tests. Responses to “How bubbly/gassy is this drink?” for each glass condition were used as a manipulation check. Outliers were detected based on likeability scores via boxplots and defined as 1.5 times the interquartile range above quartile 3 or below quartile 1. All analyses were conducted using SPSS (International Business Machines (IBM) SPSS Statistics for Windows, Version 23.0, IBM Corporation). This analysis plan was preregistered on the Open Science Framework.

The data that form the basis of the results presented here and the analysis scripts used to generate them are available from the University of Bristol Research Data Repository (http://data.bris.ac.uk/data/), doi: 10.5523/bris.11gvdrsp5sxo72l6s0suq89e4a.

### Study 2

#### Design and overview

This was a laboratory experimental study. We used a between-subjects, double-blind design with glass (nucleated, non-nucleated) as the between-subjects factor. The protocol was registered at http://osf.io/rcmuj prior to data collection.

#### Participants

Identical criteria were used to select participants as in Study 1, with an additional exclusion criterion of not having taken part in Study 1 or a previous experiment investigating the effect of glass markings on drinking rate. Participants were reimbursed £7 or awarded course credit at the end of the study.

#### Randomisation

Block randomisation was used to allocate equal number of participants to glass conditions (nucleated, non-nucleated) stratified by sex. Random number assignment software randomised participants to glass conditions.

#### Materials

**Fig. 2:**
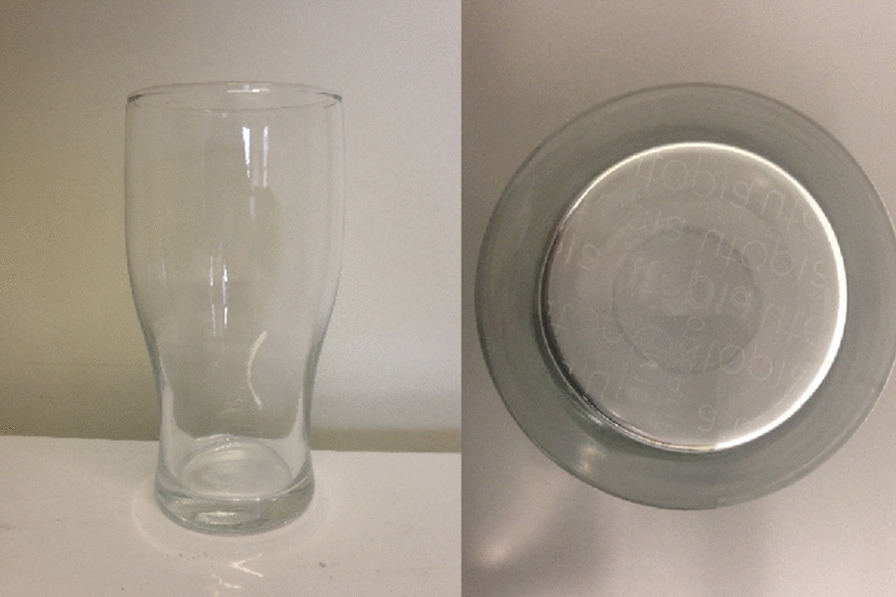
Tulip beer glass (left) and its nucleated base (right)

The same alcoholic beverage was used as in Study 1 with a slightly different ABV of 5%. Glassware used were tulip shaped beer glasses (volume: 568 ml; Fig. 2) supplied by Paşabahçe*.* One was a “Head Keeper” nucleated glass, and the other was non-nucleated. Glasses were otherwise identical.

Questionnaire measures used were identical to Study 1 with some additions. The Alcohol Urges Questionnaire (AUQ) [32] was administered to assess craving for alcohol as this may influence consumption as scores on the AUQ have been shown to have positive associations with drinks per week [33]. The Positive and Negative Affect Schedule (PANAS) [34] was administered to assess mood as it has been shown that mood affects drinking motives and fluctuates during drinking episodes [35]. The National Adult Reading Test (NART) [36] and an online word-search task were also included as dummy tasks.

#### Procedure

Participants attended one study session lasting approximately 45 min. Participants underwent the same pre-experiment procedure and screening as Study 1 except for being told that the aim of the study was to measure the effects of alcohol consumption on word-search task performance, in order to minimise demand characteristics.

In the main session, participants were asked to turn their phone off and place it out of reach. They were presented with 100 ml of water as a thirst quencher and consumed as much as they liked. Self-reported measures of alcohol use (AUDIT), alcohol craving (AUQ) and mood (PANAS) were administered. Participants completed the NART and then received 568 ml of lager (5% ABV Budweiser in a nucleated or non-nucleated glass as per randomisation). Drinks were prepared as per Study 1. Participants were told that they should consume all of the drink at their own pace whilst watching a nature documentary (Earth: The Journey of a Lifetime, British Broadcasting Corporation Worldwide 2008). The experimenter started the film (at the same point in the film and in the session for all participants) and left the room. The drinking session was recorded using a video camera (Hitachi Hybrid Camcorder DZHS500E) to allow extraction of drinking times. Participants opened the door when they had finished their beverage, the experimenter returned and presented participants with the LDEQ and an online word-search task in which they were instructed to find as many words as possible in four minutes. This was intended to disguise the nature of the study, and these data were not analysed. Then, measures of alcohol craving (AUQ) and mood (PANAS) were administered again. Finally, participants were debriefed and reimbursed. Before leaving the testing room, the participant underwent the same debriefing and safety protocol as Study 1.

#### Statistical analysis

A study investigating the effect of glass shape on the drinking rate of lager (no evidence from a study directly investigating nucleation was available) indicated a longer drinking time from straight glasses (*M* = 11.5, SD = 5.6) compared to curved glasses (*M* = 7.2, SD = 3.3), representing an effect size of *d* = 0.91 for the difference in drinking rate between the two glass shapes [26]. However, in order to be conservative, we recruited a sample size of 160 participants, which provided 80% power at an alpha level of 5% to detect an effect size of *d* = 0.45, which is equivalent to a difference in drinking rate of 2 min (SD = 4.5) between conditions.

Questionnaire responses were captured via online survey platforms (Bristol Online Survey & Qualtrics) and imported into SPSS. Drinking time data was extracted from videos. The primary outcome measure was total drink time (from initiation of first sip to termination of last sip), and we analysed these data in a linear regression, with glass type (nucleated, non-nucleated) as a between-subjects factor. Outliers were detected based on total drinking times via boxplots and defined the same as in Study 1. The secondary outcome of likeability of lager was assessed by calculating likeability scores as in Study 1 and individual questionnaire items that constituted it were analysed using independent samples *t*-tests, with glass type as a between-subjects factor. The secondary outcomes of mood and alcohol craving were assessed using linear regressions with glass type as predictor adjusting for baseline mood/craving to analyse mood (PANAS) and craving (AUQ) data respectively. Responses to the question “How bubbly/gassy was the drink?” served as a manipulation check and was analysed using an independent samples *t* test, with glass type as a between-subjects factor. All analyses were conducted using SPSS (IBM SPSS Statistics for Windows, Version 23.0, IBM Corporation). This analysis plan was preregistered on the Open Science Framework.

The data that form the basis of the results presented here and the analysis scripts used to generate them are available from the University of Bristol Research Data Repository (http://data.bris.ac.uk/data/), doi: 10.5523/bris.lxp8eavh7wnn2p57q98rcn2go.

## Results

### Study 1

Participants (*n* = 116) were on average 21 years old (standard deviation [SD] = 4, range 18–37) with an AUDIT score of 10 (SD = 4, range 4–26). 27% of participants were low-risk drinkers (AUDIT score: 0–7), 64% were hazardous drinkers (AUDIT score: 8–15) and 10% were harmful drinkers (AUDIT score: 16 +). When asked what drink they preferred, 54% of participants chose the nucleated lager. Four extra participants were tested than planned to balance the number of participants in each condition after a randomisation error occurred during testing.

#### Manipulation check

A paired-samples *t*-test found strong evidence for a difference in the nucleated compared to the non-nucleated condition, suggesting that lager in nucleated glasses was more bubbly/gassy compared to lager in non-nucleated glasses. Removing outliers (*n* = 2) did not meaningfully change the results. These results suggest the experimental manipulation worked as intended (Table 1).

**Table 1:** Differences in likeability (including sub-scales) of lager and volume of lager consumed between conditions

	Full sample (*n* = 116)			Outliers excluded (*n* = 114)		
	MD	95% CI	*p* value	MD	95% CI	*p-*value
Total (likeability) score	0.7	− 2.4, 3.9	0.64	0.7	− 2.4, 3.8	0.66
*Likeability sub-scales*						
Visual appeal	9.3	5.0, 13.6	< 0.001	10.2	6.1, 14.2	< 0.001
Enjoyment	2.1	− 2.0, 6.2	0.31	2.2	− 2.0, 6.4	0.29
Refreshment	3.3	− 0.6, 7.2	0.10	3.4	− 0.6, 7.4	0.09
Tastiness	0.3	− 4.1, 4.7	0.88	0.4	− 4.1, 4.9	0.87
Likelihood to buy	− 0.1	− 4.9, 4.7	0.96	− 0.1	− 4.9, 4.8	0.98
*Other items*						
Bubbly/gassy	13.9	8.9, 18.9	< 0.001	14.1	9.0, 19.1	< 0.001
Volume consumed (ml)	0.4	− 9.5, 10.4	0.93	0.7	− 9.4, 10.7	0.90

*CI* confidence interval, *MD* mean difference

#### Likeability factor (primary outcome)

We found no clear evidence for a difference in overall likeability of lager from a nucleated glass and a non-nucleated glass, but we found strong evidence for a difference in visual appeal of lager consumed from a nucleated glass compared to a non-nucleated glass. There was no clear evidence to suggest meaningful differences in responses to the other three questions constituting the likeability factor. Removing outliers (*n* = 2) did not change any of these effects meaningfully (Table 1/Additional file 1: Table S1).

#### Volume consumption (secondary outcome)

We found no clear evidence for a difference in the volume of lager consumed from a nucleated glass (Mean [*M*] = 183.5 ml [SD = 75.5]) and a non-nucleated glass (*M* = 183.1 ml [SD = 75.6]). Removing outliers (*n* = 2) did not alter these results meaningfully (Table 1). Means and standard deviations can be found in Additional file 1: Table S1.

### Study 2

Participants (*n* = 160; 50% female) were on average 21 years (SD = 4, range 18–40) and had an average AUDIT score of 9 (SD = 4, range 2–22). These characteristics were similar across groups (nucleated, non-nucleated) with an average age of 21 years (nucleated: SD = 4, range 18–34; non-nucleated: SD = 4, range 18–40; *t*(158) = − 0.167, *p* = 0.868) and average AUDIT scores of 9 (nucleated: SD = 4, range 2–22; non-nucleated: SD = 3, range 3–18; *t*(158) = − 0.188, *p* = 0.851) in both groups. 45% of participants were low-risk drinkers (AUDIT score: 0–7), 49% were hazardous drinkers (AUDIT score: 8–15) and 6% were harmful drinkers (AUDIT score: 16 +). Two extra participants were tested to replace two participants excluded from analysis due to video malfunctions making their data unusable. Missing questionnaire responses were imputed based on the median of the sample for that specific question. Five outliers were removed based on their drinking time using the same exclusion criterion as Study 1.

#### Total drinking time (primary outcome)

There was no clear evidence that nucleated glasses were associated with total drinking time in the full sample (MD = 1.5, 95% CI − 1.0 to 4.0, *p* = 0.25) or when outliers (*n* = 5) were removed (MD = 0.6, 95% CI − 1.5 to 2.7, *p* = 0.57). Means and standard deviations can be found in Additional file 1: Table S5.

#### Reliability analysis

Ratings of total drinking time carried out by two raters were strongly and positively correlated, with single measures intraclass correlation indicating a high level of inter-rater reliability (*rs* > 0.96, *ps* < 0.001).

#### Likeability factor (secondary outcome)

An independent samples *t*-test found no clear evidence for a difference between the likeability of lager from a nucleated glass and a non-nucleated glass (Table 2). Removing five outliers did not meaningfully change the results. There was no clear evidence to suggest differences in responses to the five questions constituting the likeability factor. Means and standard deviations can be found in Additional file 1: Table S4.

**Table 2:** Differences in likeability of lager (including sub-scales) between nucleated and non-nucleated conditions

	Full sample (*n* = 160)			Outliers excluded (*n* = 155)		
	MD	95% CI	*p* value	MD	95% CI	*p* value
Total (likeability) score	− 0.7	− 6.6, 5.2	0.82	− 0.2	− 6.1, 5.8	0.96
*Likeability sub-scales:*						
Visual appeal	− 1.6	− 8.5, 5.3	0.64	− 0.3	− 7.3, 6.6	0.93
Enjoyment	− 2.3	− 9.1, 4.6	0.52	− 1.5	− 8.3, 5.3	0.67
Refreshment	− 3.2	− 9.2, 2.9	0.30	− 3.5	− 9.6, 2.7	0.27
Tastiness	1.2	− 5.4, 7.9	0.71	1.4	− 5.3, 8.1	0.69
Likelihood to buy	2.3	− 5.2, 9.8	0.54	3.1	− 4.3, 10.5	0.41
*Other items*						
Bubbly/gassy	− 0.8	− 6.6, 5.0	0.78	− 0.3	− 6.3, 5.6	0.91

*MD* mean difference, *CI* confidence interval

#### AUQ (secondary outcome)

There was no clear evidence that glass type predicted the change in alcohol craving when controlling for baseline alcohol craving in the full sample (MD = − 0.1, 95% CI − 0.4 to 0.2, *p* = 0.65) and when outliers (*n* = 5) were removed (MD = − 0.04, 95% CI − 0.3 to 0.3, *p* = 0.83, Additional file 1: Table S2). Means and standard deviations can be found in Additional file 1: Table S3.

#### PANAS (secondary outcome)

There was no clear evidence that glass type predicted the change in positive affect when controlling for baseline positive affect in the full sample (MD = − 1.0, 95% CI − 2.5 to 0.5, *p* = 0.17) or when outliers (*n* = 5) were removed (MD = − 1.1, 95% CI − 2.6 to 0.4, *p* = 0.16, Additional file 1: Table S4). There was no clear evidence that glass type predicted the change in negative affect when controlling for baseline negative affect in the full sample (MD = − 0.4, 95% CI − 1.0 to 0.2, *p* = 0.22) or when outliers (*n* = 5) were removed (MD = − 0.3, 95% CI − 1.0 to 0.3, *p* = 0.30, Additional file 1: Table S2). Means and standard deviations can be found in Additional file 1: Table S3.

#### Manipulation check

There was no clear evidence in the full sample or when outliers (*n* = 5) were removed (Table 2) for a difference in responding to the question “How bubbly/gassy was the drink?”.

## Discussion

Contrary to our hypotheses, nucleated glassware did not alter the overall likeability of lager in either study. In Study 1, there was strong evidence (*p* < 0.001) that the visual appeal of lager was greater and weak evidence (*p* = 0.09) that refreshment was greater when consuming from nucleated compared to non-nucleated glasses. Nucleated glasses did not appear to affect lager consumption in terms of volume consumed in a set time period of five minutes (Study 1) or drinking rate (Study 2).

In support of the findings of Study 1 on visual appeal, participants in other studies have been observed paying attention to the continuous flow of ascending bubbles during champagne and sparkling wine tasting and noting their visual appeal [37]. Similarly, a medium level of beer “head” foam has been judged the most visually appealing by both males and females [10]. The effervescent effect of ascending bubbles and beer “head” which can be maintained for longer in nucleated glasses appears to be visually appealing to drinkers. The perception of nucleated lagers being more refreshing than non-nucleated lagers could be the result of dissolved CO_2_ and collapsing bubbles in the oral cavity interacting with trigeminal receptors which are responsible for face sensations [17, 18] and gustatory receptors which are responsible for taste sensations [19, 20] as is the case when sparkling wines or champagne are consumed. There was no clear evidence for a difference in any of the five individual questionnaire items that constituted the likeability factor in Study 2. A possible explanation could be the difference in perceived effect of nucleation in both studies possibly caused by the change in glassware. Participants rated lager in nucleated glasses as being more bubbly/gassy than lager in non-nucleated glasses in Study 1 but not in Study 2. This could be due to the different time spent consuming beverages in both studies (i.e. 5 min in Study 1, average 17 min in Study 2). The effect of nucleation does diminish over time; therefore, participants in Study 2 would have observed the lager being less bubbly/gassy in the nucleated condition than participants in Study 1. Additionally, the LDEQ was administered post-consumption in Study 2 relying on participant recall of how bubbly/gassy the lager was during consumption introducing possible recency bias and memory artefacts.

The perceived increase in visual appeal and refreshment in nucleated glasses in Study 1 did not lead to a meaningful difference in volume consumed. There was also no meaningful difference in drinking rate in Study 2. This suggests that nucleation cannot reduce harm directly through a reduction in demand or reduced intoxication through the slowing of alcohol consumption. However, it is plausible that nucleated glasses that increase the carbonation of lager could lead to faster rates of alcohol absorption. It has been shown in previous work that adding a carbonated mixer to vodka has resulted in more participants absorbing alcohol at a faster rate compared to when no mixer or an non-carbonated mixer was used [38]. This hypothesis should be tested involving lager products in future as it may contribute to the social harms of excessive alcohol consumption due to increased intoxication if nucleated glasses are found to increase rates of alcohol absorption.

It has been suggested that the intention of nucleating glassware is to replenish and maintain the head of foam during the consumption of beer [39]. It is plausible that the nucleating of glassware is primarily focused at improving the aesthetics and refreshment of lager, which we saw some evidence for in Study 1. Nucleation may be a tool used by the alcohol industry to make their products more attractive and enticing to consumers to increase market share and instil brand loyalty whilst not explicitly attempting to change how the product is consumed. This in turn may lead the consumer to participate in more drinking episodes which would likely increase their alcohol related harm. These findings suggest that removing nucleated glasses from circulation would decrease the visual appeal and refreshment of lager products and possibly reduce the demand from consumers for these products. This may reduce the frequency of which these products are consumed and/or number of drinking episodes involving these products which could reduce the social and health harms associated with excessive alcohol consumption. Future research should investigate if by making lager products less visually appealing and refreshing by removing nucleated bases, can consumption and purchasing behaviours be changed over time in real-world settings.

Some limitations should be considered when interpreting these findings. First, in Study 2, the experimental manipulation may not have had the planned effect of altering nucleation. We can infer this from the fact that there was no evidence for a difference in the perception of how bubbly/gassy lagers were in the two glasses in Study 2, and nucleation was not directly measured. Therefore, results may not represent the true influence of nucleation on drinking rate. The different timepoints of when ratings of how bubbly/gassy the lagers were taken in both studies (during consumption in Study 1 and after consumption in Study 2) may explain the difference in ratings between studies given the effects of nucleation dissipates over time. Future studies could address this by taking multiple measures of how nucleation is perceived across the drinking episode. Furthermore, the disruption of filling out a questionnaire during consumption may have reduced the volume consumed compared to naturalistic drinking and may have caused the drinker to be more attentive to the drinking experience than they would be in a real-world drinking environment, further reducing the generalisability of the results of Study 1. Second, both studies were carried out in a laboratory setting in a predominantly university student sample and findings may not generalise to naturalistic environments and to other populations. Particularly in Study 1, a small amount of lager was consumed (~ 180 ml) compared to typical drinking settings where drinkers are likely to be consuming much larger amounts over multiple drinks. Further research can be conducted in naturalistic environments in more general populations to ascertain real-world effects of nucleation on lager likeability and consumption. Finally, the likeability questionnaire used in both studies was not a validated measure of likeability of lager and its construct validity is unknown. Future work should focus on validating a scale that assesses the likeability of lager.

## Conclusions

In conclusion, there was no meaningful difference in overall likeability of lager consumed from nucleated and non-nucleated glasses in either study. In Study 1, lager in nucleated glasses was rated as more visually appealing and refreshing than lager in non-nucleated glasses, however this was not replicated in Study 2 with pint-sized glasses. By making lager products less visually appealing and refreshing by removing nucleated bases, consumers may be less likely to purchase these products and choose to consume them in fewer drinking episodes. Reducing demand for alcohol products in this way may be the most promising avenue to reduce alcohol harm in the context of nucleation. Purchasing behaviours need to be investigated in naturalistic drinking environments to test the hypothesis that the removal of nucleation can lead to a reduction in demand for alcohol products. Based on consumption findings in both studies, keeping in mind the limitations in study design and setting that limit the generalisability of findings to real-world drinking scenarios, nucleation does not appear to have potential as a target for harm reduction in terms of reducing or slowing consumption. However, future research should investigate the effect of nucleated glasses on the volume consumed or drinking rate of alcoholic beverages over longer drinking periods and across multiple drinks and determine if the null findings are replicated across studies.

## Supplementary Information

**Additional file 1**. Description of data: Additional means and standard deviations of outcomes in both studies.

## Data Availability

The datasets supporting the conclusions of this article are available in the University of Bristol data repository.

## Abbreviations

CO_2_: Carbon dioxide
ml: Millilitre
VAS: Visual analogue scale
MD: Mean difference
CI: Confidence interval
kg: Kilogram
ABV: Alcohol by volume
AUDIT: Alcohol Use Disorder Identification Test
LDEQ: Lager Drinking Experience Questionnaire
mm: Millimetre
SPSS: Statistical Package for the Social Sciences
IBM: International Business Machines
SD: Standard deviation
M: Mean
AUQ: Alcohol Urges Questionnaire
PANAS: Positive and Negative Affect Schedule
NART: The National Adult Reading Test
