# Supplementary material for: Nucleation increases the visual appeal of lager but does not alter overall likeability or drinking rate
Source: Harm Reduct J. 2022 Apr 20;19:39. doi: 10.1186/s12954-022-00618-4 (PMC9022288; doi:10.1186/s12954-022-00618-4)
Supplement: Supplementary file 1 — Additional file 1. Description of data: Additional means and standard deviations of outcomes in both studies. [file 12954_2022_618_MOESM1_ESM.docx]

Table 1. Differences in likeability (including sub-scales) of lager and volume of lager consumed between conditions (Study 1).

|  | Full sample (*n* = 116) | | Outliers excluded (*n* = 114) | |
| --- | --- | --- | --- | --- |
|  | Nuc Mean (SD) | Non-nuc Mean (SD) | Nuc Mean (SD) | Non-nuc Mean (SD) |
| Total (Likeability) Score | 63.2 (13.9) | 62.5 (14.0) | 63.8 (13.0) | 63.1 (13.4) |
| *Likeability sub-scales:* | | | | |
| Visual Appeal | 73.6 (17.9) | 64.3 (20.0) | 74.2 (17.1) | 64.0 (20.0) |
| Enjoyment | 70.0 (17.9) | 67.9 (18.3) | 70.5 (17.3) | 68.2 (18.0) |
| Refreshment | 70.9 (16.8) | 67.6 (18.7) | 71.5 (16.0) | 68.0 (18.2) |
| Tastiness | 61.9 (20.1) | 61.6 (19.9) | 62.4 (19.5) | 62.0 (19.3) |
| Likelihood to Buy | 62.8 (24.3) | 62.9 (22.3) | 63.4 (23.8) | 63.4 (21.7) |
| *Other items:* | | | | |
| Bubbly / gassy | 72.4 (20.1) | 58.5 (23.6) | 72.4 (20.1) | 58.3 (23.7) |
| Volume Consumed (ml) | 183.5 (75.5) | 183.1 (75.6) | 184.7 (74.7) | 184.0 (75.5) |

Nuc = Nucleated condition. Non-nuc = Non-nucleated condition. SD = Standard Deviation.

Table 2. Effect of nucleation on post-drinking alcohol craving and mood (*Study 2*).

| Adjusted for baseline | | |  |  |
| --- | --- | --- | --- | --- |
|  | | MD | 95% CI | *p*-value |
| Full sample (n = 160) | AUQ | -0.1 | -0.4, 0.2 | 0.65 |
|  | PANAS positive | -1.0 | -2.5, 0.5 | 0.17 |
|  | PANAS negative | -0.4 | -1.0, 0.2 | 0.22 |
| Outliers excluded (n = 155) | AUQ | -0.04 | -0.3, 0.3 | 0.83 |
|  | PANAS positive | -1.1 | -2.6, 0.4 | 0.16 |
|  | PANAS negative | -0.3 | -1.0, 0.3 | 0.30 |

MD = Mean Difference. AUQ = Alcohol Urges Questionnaire. PANAS = Positive and Negative Affect Schedule.

Table 3. Effect of nucleation on post-drinking alcohol craving and mood (Study 2).

| Nucleated (SD) | | Non-nucleated (SD) | |  |  |
| --- | --- | --- | --- | --- | --- |
|  | | Time 1 | Time 2 | Time 1 | Time 2 |
| Full sample (n = 160) | AUQ | 2.4 (1.1) | 2.6 (1.2) | 2.2 (0.9) | 2.5 (0.9) |
|  | PANAS positive | 28.1 (5.8) | 26.3 (8.3) | 26.9 (7.6) | 26.1 (8.1) |
|  | PANAS negative | 12.3 (3.3) | 11.3 (2.1) | 12.5 (3.0) | 11.8 (3.1) |
| Outliers excluded (n = 155) | AUQ | 2.4 (1.1) | 2.6 (1.2) | 2.2 (0.9) | 2.6 (0.9) |
|  | PANAS positive | 28.2 (5.9) | 26.5 (8.4) | 26.8 (7.6) | 26.2 (8.1) |
|  | PANAS negative | 12.3 (3.3) | 11.3 (2.1) | 12.5 (3.0) | 11.8 (3.1) |

SD = Standard Deviation. AUQ = Alcohol Urges Questionnaire. PANAS = Positive and Negative Affect Schedule.

Table 4. Differences in likeability (including sub-scales) of lager between nucleated and non-nucleated conditions (Study 2).

|  | Full sample (*n* = 160) | | Outliers excluded (*n* = 155) | |
| --- | --- | --- | --- | --- |
|  | Nuc Mean (SD)  *n* = 80 | Non-nuc Mean (SD)  *n* = 80 | Nuc Mean (SD)  *n* = 76 | Non-nuc Mean (SD)  *n* = 79 |
| Total (Likeability) Score | 62.8 (19.3) | 63.5 (18.5) | 63.7 (19.1) | 63.8 (18.4) |
| *Likeability sub-scales:* | | | | |
| Visual Appeal | 63.3 (22.3) | 65.0 (21.7) | 64.6 (21.9) | 64.9 (21.8) |
| Enjoyment | 63.1 (22.5) | 65.3 (21.3) | 64.2 (21.9) | 65.7 (21.1) |
| Refreshment | 65.3 (20.7) | 68.5 (17.8) | 65.5 (21.1) | 68.9 (17.5) |
| Tastiness | 60.8 (21.1) | 59.6 (21.3) | 61.4 (21.1) | 60.0 (21.1) |
| Likelihood to Buy | 61.4 (22.8) | 59.1 (24.9) | 62.6 (21.9) | 59.5 (24.7) |
| *Other items:* | | | | |
| Bubbly / gassy | 70.5 (18.6) | 71.4 (18.6) | 70.8 (18.9) | 71.1 (18.7) |

Nuc = Nucleated condition. Non-nuc = Non-nucleated condition. SD = Standard Deviation.

Table 5. Effect of nucleation on total drinking time (min:sec/0.6; Study 2).

|  | Mean drinking time | |
| --- | --- | --- |
|  | Nucleated (SD) | Non-nucleated (SD) |
| Full sample (n = 160) | 18.2 (8.7) | 16.7 (7.2) |
| Outliers excluded (n = 155) | 16.9 (6.9) | 16.3 (6.3) |

SD = Standard Deviation.
